# Microperimetric evaluation and predictive factors of visual recovery after successful inverted internal limiting membrane-flap technique for macular hole in high myopic eyes

**DOI:** 10.3389/fmed.2023.1276502

**Published:** 2023-11-23

**Authors:** Alessandra Sborgia, Giacomo Boscia, Alfredo Niro, Luca Landini, Valentina Pastore, Valeria Albano, Marina Piepoli, Rossella Donghia, Stefano Dore, Pasquale Viggiano, Rosa Buonamassa, Camilla Di Pardo, Teresa Molfetta, Eye Clinic Research Group, Antonella Guglielmi, Giacomo Scotti, Marida Gaudiomonte, Roberto Semeraro, Marco Coassin, Roberto Dell’Omo, Francesco Boscia, Giovanni Alessio, Giancarlo Sborgia

**Affiliations:** ^1^Department of Basic Medical Sciences, Neuroscience and Sense Organs, University of Bari “Aldo Moro”, Bari, Italy; ^2^Eye Clinic Section, Department of Surgical Sciences, University of Turin, Turin, Italy; ^3^Eye Clinic, “SS. Annunziata” Hospital, ASL Taranto, Taranto, Italy; ^4^National Institute of Gastroenterology "S. de Bellis" Research Hospital, Castellana Grotte, Bari, Italy; ^5^Department of Medical, Surgical and Experimental Sciences, University of Sassari, Sassari, Italy; ^6^Ophthalmology, University Campus Bio Medico of Rome, Roma, Italy; ^7^Department of Medicine and Health Sciences “Vincenzo Tiberio”, University of Molise, Campobasso, Italy

**Keywords:** high myopia, macular hole, inverted ILM-flap, microperimetry, retinal sensitivity, fixation behavior, axial length

## Abstract

**Introduction:**

Inverted Internal Limiting Membrane (ILM)-flap technique demonstrated its effectiveness, in terms of anatomical closure rate and visual acuity recovery for high myopic macular holes. We evaluated macular function after a successful inverted ILM-flap for macular holes in high myopic eyes (hMMH) using microperimetry to predict visual prognosis.

**Methods:**

A retrospective study on 23 eyes of 23 patients after surgical closure of hMMH, was performed. All patients underwent inverted ILM-flap and gas tamponade. Cataract surgery was performed in phakic eyes. Study outcomes including best-corrected visual acuity (BCVA), retinal sensitivity (RS) at central 12°, central retinal sensitivity (CRS) at central 4° and mean deviation (MD), and fixation behavior as bivariate contour ellipse area (BCEA, degrees2) measured by microperimetry, were evaluated over 6 months. A mixed-effects model was used to evaluate and compare the repeated measurements of outcomes between phakic and pseudophakic eyes. A regression model was performed to assess the relationship between BCVA at 6 months and independent variables.

**Results:**

Overall mean BCVA improved from 0.98 ± 0.21 logMAR at baseline to 0.47 ± 0.31 logMAR at the last follow-up (*p* < 0.001). Over 6 months, overall sensitivity measurements improved (RS, *p* = 0.001; CRS, *p* < 0.0001; MD, *p* = 0.03), and the BCEA decreased in dimension, although not significantly (*p* ≥ 0.05). The mixed model revealed a significantly better effect of inverted ILM-flap combined with cataract surgery on BCVA and CRS in phakic eyes than inverted ILM-flap alone in pseudophakic ones. The regression model revealed a relationship of 6-month BCVA with pre-operative BCVA (*β* = 0.60, *p* = 0.02) and RS (*β* = −0.03, *p* = 0.01).

**Conclusion:**

The inverted ILM-flap technique significantly improved visual acuity and retinal sensitivity after the hMMH closure, particularly when combined with cataract extraction. Pre-operative visual acuity and retinal sensitivity at central 12° may predict post-surgical visual acuity.

## Introduction

Macular hole (MH) is a known clinical finding in patients with high myopia (1), with a prevalence of 8.5% (2). The age at the onset of MH in highly myopic eyes (hMMH) significantly decreases with the increase of myopic refraction (1).

Several factors, including myopic maculopathy traction (2), epiretinal membrane, the rigidity of the internal limiting membrane (ILM) and retinal vessels, may promote the development of hMMH (3).

Jo et al. (4) recommended surgical intervention when macular traction and visual acuity impairment progresses. However, in a small percentage (6.3%) of cases, hMMH may be asymptomatic and only be revealed by optical coherence tomography (OCT) scans (5).

Surgical procedures on ILM, such as the traditional ILM peeling, autologous transplantation of ILM, and the inverted ILM-flap, have been successfully used to decrease tractional forces (6). In particular, the inverted ILM-flap demonstrated equal and sometimes greater effectiveness in terms of anatomical closure rate and visual acuity recovery when compared to other techniques (7–9).

The analysis of only visual acuity partially reveals the macular function’s complexity. In recent years, there has been an increase in the use of fundus-related microperimetry that can assess macular function by simultaneously imaging the fundus and projecting light stimuli onto a testing point. Furthermore, microperimetry revealed its more sensitive to a macular functional deficit than visual acuity (10). Using microperimetry as a functional assay helped evaluate changes in retinal function among patients with myopic maculopathy. The microperimeric analysis revealed that retinal sensitivity was strongly associated with the retinal microstructural changes according to the severity of myopic degeneration (11).

Microperimetry has provided quantitative measures of macular function, such as retinal sensitivity and fixation behavior, both before and after macular surgery. This has been observed in various conditions, including hMMH (12–17). Furthermore, pre-operative retinal sensitivity at central degrees and fixation behavior have already been demonstrated to predict post-surgical visual acuity for large idiopathic MHs (16).

We evaluated the changes in visual and microperimetric outcomes after the surgical success of the inverted ILM-flap technique for hMMH to predict visual prognosis.

## Materials and methods

We conducted a retrospective, single-center cohort study on patients who underwent a successful inverted ILM-flap approach for hMMH. All patients were referred to the Ophthalmology Clinic of the University of Bari, Italy. The study was approved by the Institutional Review Board (IRB; date: 09 January 2019, Eye Clinic, Department of Medical Science, Neuroscience and Sense Organs, University of Bari, Bari, Italy) and adhered to the tenets of the Declaration of Helsinki. All participants read and signed written informed consent.

Enrolled subjects were 18 years old or older, with high myopia, defined as axial length greater than 26 mm [measured with Zeiss IOLMaster 500^®^ (SNR > 200)] or a myopic refractive error of ≥ − 6.0 diopters (3, 17), and a feature of closed full-thickness MH after vitrectomy with Inverted Internal Limiting Membrane (ILM)-flap technique, as revealed by OCT scans (18).

Exclusion criteria were amblyopia, corneal disease, a subcortical cataract or cataract of more than 3 nuclear sclerosis or cortical opacity, glaucoma or ocular hypertension, previous vitreoretinal surgery, diabetic retinopathy, retinal vascular disease, age-related macular degeneration, idiopathic or traumatic MHs, myopic foveoschisis with or without foveal detachment, MH complicated by a retinal detachment, and a minimum diameter of MH >1,000 μm, and the presence of a patchy chorioretinal atrophy involving the fovea [diagnosis based on spectral-domain optical coherence tomography (SD-OCT), showing light backscattering and the absence of outer retinal layers around the MH].

### Assessment

Each patient underwent a complete ophthalmic examination, including best corrected visual acuity (BCVA) measurement using ETDRS charts, slit lamp biomicroscopy, indirect ophthalmoscopy, OCT, and microperimetry. BCVA was recorded with Early Treatment Diabetic Retinopathy Study (ETDRS) chart at 4 meters. ETDRS values were converted to the logarithm of the minimum angle of resolution (logMAR) for statistical analysis. OCT was performed with Spectralis OCT (Spectralis HRA + OCT, Heidelberg Engineering, Heidelberg, Germany) using Star Scans (six sections, 20, 512 A scan) and vertical and horizontal line (30, 768-A scans) scans passing through the fovea.

Retinal sensitivity and fixation behavior were evaluated by an MP-1 microperimeter (MP-1, Nidek Technologies, Padua, Italy). Retinal sensitivity was measured across a 45-point grid centered on the fovea. Sensitivity was measured using a white stimulus 0.4 degrees in diameter presented for 200 ms against a mesopic background. The threshold at each point was determined using a 4–2 staircase. The “follow-up” feature of the microperimeter was used to obtain measurements at the same retinal sites during overall visits. Mean retinal sensitivity (RS), the mean sensitivity of all 45 loci in the central 12° (1 = 300 μm), and mean central retinal sensitivity (CRS), the mean sensitivity of the central 13 loci (enclosed by a circle with a 4° diameter) were recorded. The software calculated the mean deviation (MD) after comparing the measured retinal sensitivity at the central 12° with a normative database derived from 180 healthy volunteers stratified into six age groups (19). During the sensitivity examination, fixation stability was also recorded (16). To calculate the average eye movement during fixation, BCEA (bivariate contour ellipse area) was used. This involves plotting the position of each fixation on Cartesian axes and determining the area of an ellipse that encloses a given percentage of fixation points. The value of standard deviations of horizontal and vertical eye movements during fixation were used to measure BCEA. We have analyzed BCEA for 68.2, 95.4, and 99.6% of fixation points (20). Each examination was performed before, at months 1,3, and 6 after surgery.

### Surgical procedure

All surgeries were performed by the same well-experienced retinal specialist (GS) under a retrobulbar block (a mixture of 2% Lidocaine and 2% Mepivacaine).

Standard cataract phacoemulsification and intraocular lens implant were performed in phakic eyes at the time of vitrectomy. A 27-Gauge sutureless vitrectomy system was used to perform a core vitrectomy. The vitreous cortex adhering to the retinal surface was removed after injection of an ophthalmic suspension containing 4% triamcinolone acetonide (Vitreal S, Fidia Farmaceutici S.p.a., Abano Terme, Italy) to visualize the vitreous. An intraocular dye composed of soluble lutein, Brilliant Blue, and Trypan Blue (DOUBLEDYNE^®^, Alfa Instruments Srl, Casoria, Italy) was used to stain the ILM. The pinch and grasp technique achieved ILM peeling of at least two disk diameters around the MMH. If necessary, we adjusted the trocar size to accommodate longer forceps (Pinnacle 360° 25ga fine tip Eckardt forceps, Myopic; Synergetics, Inc., O’Fallon, MO, United States). The ILM was trimmed with the vitrector and inverted to cover the hole. A non-expansile mixture of SF6 at a concentration of 20% was injected at the end of the procedure as a tamponade, and patients were instructed to maintain a facedown position for 3 days after surgery (21).

### Statistical analysis

Statistical analysis was based on all patients included in the study. No formal sample size calculation was performed. Mean and standard deviation for continuous variables and relative frequency for categorical were used. A Friedman’s test was performed on the changes in morfunctional parameters over follow-up. A categorization of the eyes according to the lens status (phakic and pseudophakic) at baseline was performed. A linear mixed model was used to evaluate repeated measurements of BCVA, RS, CRS, MD, and BCEA at each time point within each group and among the groups, and the trajectories of BCVA, RS, CRS, MD, and BCEA.

All statistical tests were performed at the *p* < 0.05 significance level.

The relationship between BCVA at 6 months and each independent variable was analyzed using the linear regression model. The independent variables included age, sex, lens status, axial length, macular hole size, baseline mean BCVA, RS, CRS, MD, and BCEA. A backward multiple regression model with a stepwise method was performed to assess any predictive factors associated with visual acuity 6 months after surgery (cut-off removal variable, *p* ≥ 0.10). Multiple regression analyses were performed on variables that correlated significantly (*p* < 0.05) with postoperative BCVA. The factors with a value of *p* < 0.05 in the multiple models were considered potential baseline predictors.

All the statistical computations were made using StataCorp, 2015, Stata Statistical Software: Release 14. College Station, TX: StataCorp LP.

## Results

A total of 26 eyes underwent an inverted ILM-flap approach for hMMH between January 2019 and November 2021. After excluding eyes with an open hMMH after surgery (1 eye), and eyes from patients not willing or able to undergo pre-operative and post-operative microperimetry testing (2 eyes), 23 eyes of 23 patients were recruited for this analysis. The age at surgery ranged from 51 to 79 years. Axial length ranged from 26.02 to 33.29 mm, and MH minimum diameter ranged from 150 μm to 709 μm (Table 1).

**Table 1:** Demographic and clinical characteristics of patients (*n = 23*).

Parameters^*^	M ± SD or %
Age (yrs)	65.5 ± 8.2
Gender (M) (%)	14 (60.87)
AL (mm)	27.39 ± 2.05
MH minimum diameter (μm)	374.61 ± 152.93
Lens Status (%)	
Phakic	16 (69.5)
Pseudophakic	7 (30.4)

*As Mean and Standard Deviation (M ± SD) for continuous variables, and frequency and percentage (%) for categorical; AL, Axial length; MH, macular hole.

All phakic eyes had undergone cataract phacoemulsification and intraocular lens implant at the time of vitrectomy. In all eyes, the gas bubble was significantly reduced at the first follow-up revealing MH closure at OCT scans. All included patients had a month-6 follow-up. No ocular or systemic complications were observed.

### BCVA

**Figure 1:**
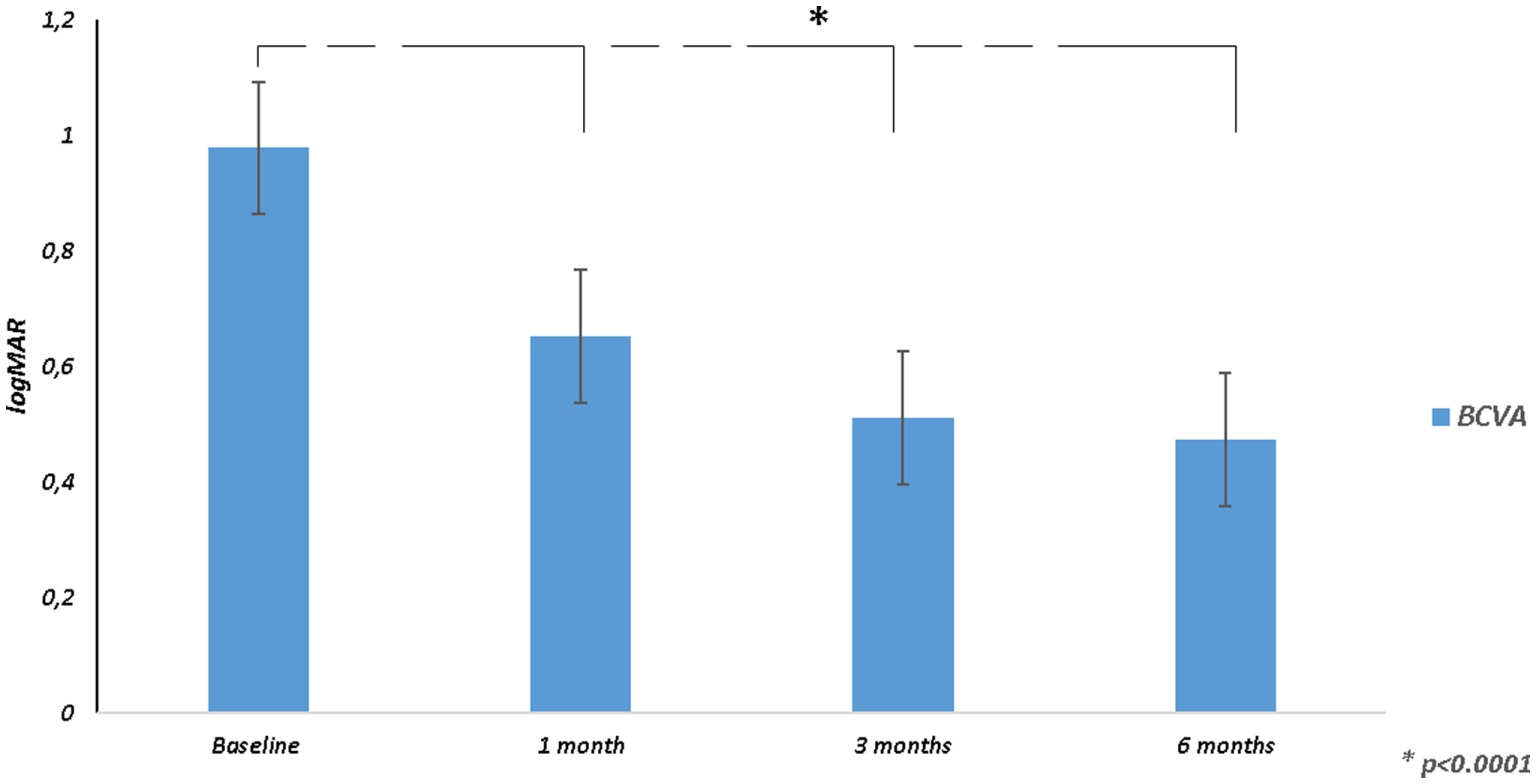
Best corrected visual acuity (BCVA) changes over follow-up.

Mean BCVA improved from 0.98 ± 0.21 logMAR at baseline to 0.47 ± 0.31 logMAR at 6 months (*p* < 0.0001; Table 2; Figure 1). All patients had an improvement in visual acuity ranging from 0.1 to 0.9 logMAR just after 1 month. Twenty-one patients had a higher visual acuity at 6 months than baseline; in only two eyes, baseline visual acuity remained stable at last follow-up (Supplementary Figure 1).

**Table 2:** Variation of functional parameters during the follow-up (*n = 23*).

Parameters^*^	Follow-up				
	Pre	1 month	3 months	6 months	*p*^
BCVA	0.98 ± 0.21	0.65 ± 0.31	0.51 ± 0.29	0.47 ± 0.31	<0.0001
RS	11.46 ± 4.91	12.23 ± 4.33	12.80 ± 4.34	13.29 ± 4.40	0.003
CRS	8.40 ± 4.76	9.38 ± 5.01	10.14 ± 4.69	11.24 ± 4.89	0.002
MD	−7.69 ± 4.64	−7.01 ± 3.97	−6.31 ± 4.07	−5.99 ± 4.28	0.03
BCEA 68.2%	5.89 ± 6.93	4.56 ± 5.23	4.12 ± 5.66	4.13 ± 5.78	0.18
BCEA 95.4%	14.30 ± 16.83	9.75 ± 8.51	9.39 ± 9.33	8.39 ± 8.17	0.15
BCEA 99.6%	21.41 ± 24.45	17.10 ± 12.12	15.44 ± 12.16	14.85 ± 10.99	0.05

*Mean and Standard Deviation (M ± SD) for continuous variables. BCVA, Best Corrected Visual Acuity; BCEA, Bivariate Contour Ellipse Area; RS, Retinal Sensitivity; MD, Mean Deviation; CRS, Central Retinal Sensitivity. ^Friedman’s test.

### Microperimetric parameters

**Figure 2:**
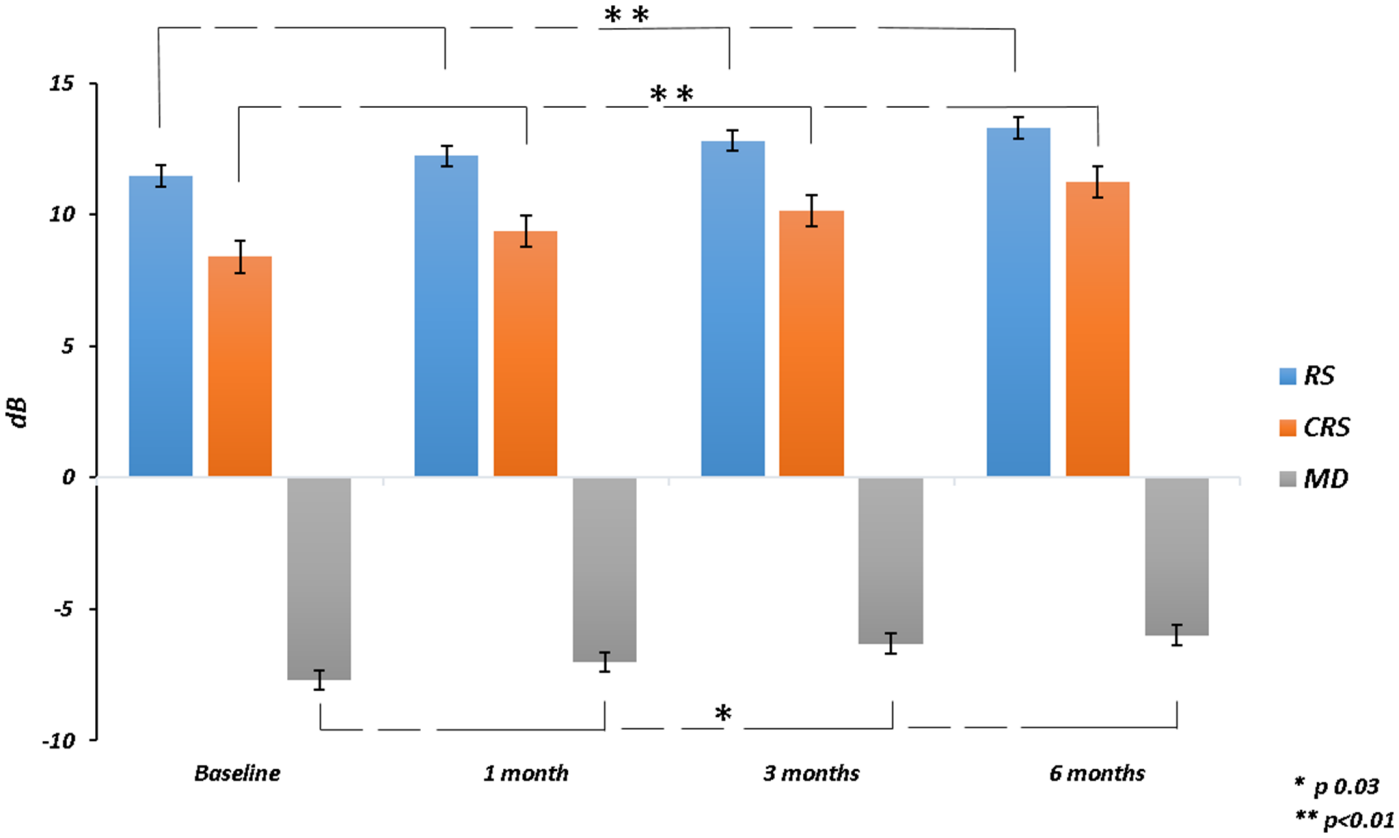
Retinal sensitivity (RS), central retinal sensitivity (CRS) and mean deviation (MD) changes over follow-up.

**Figure 3:**
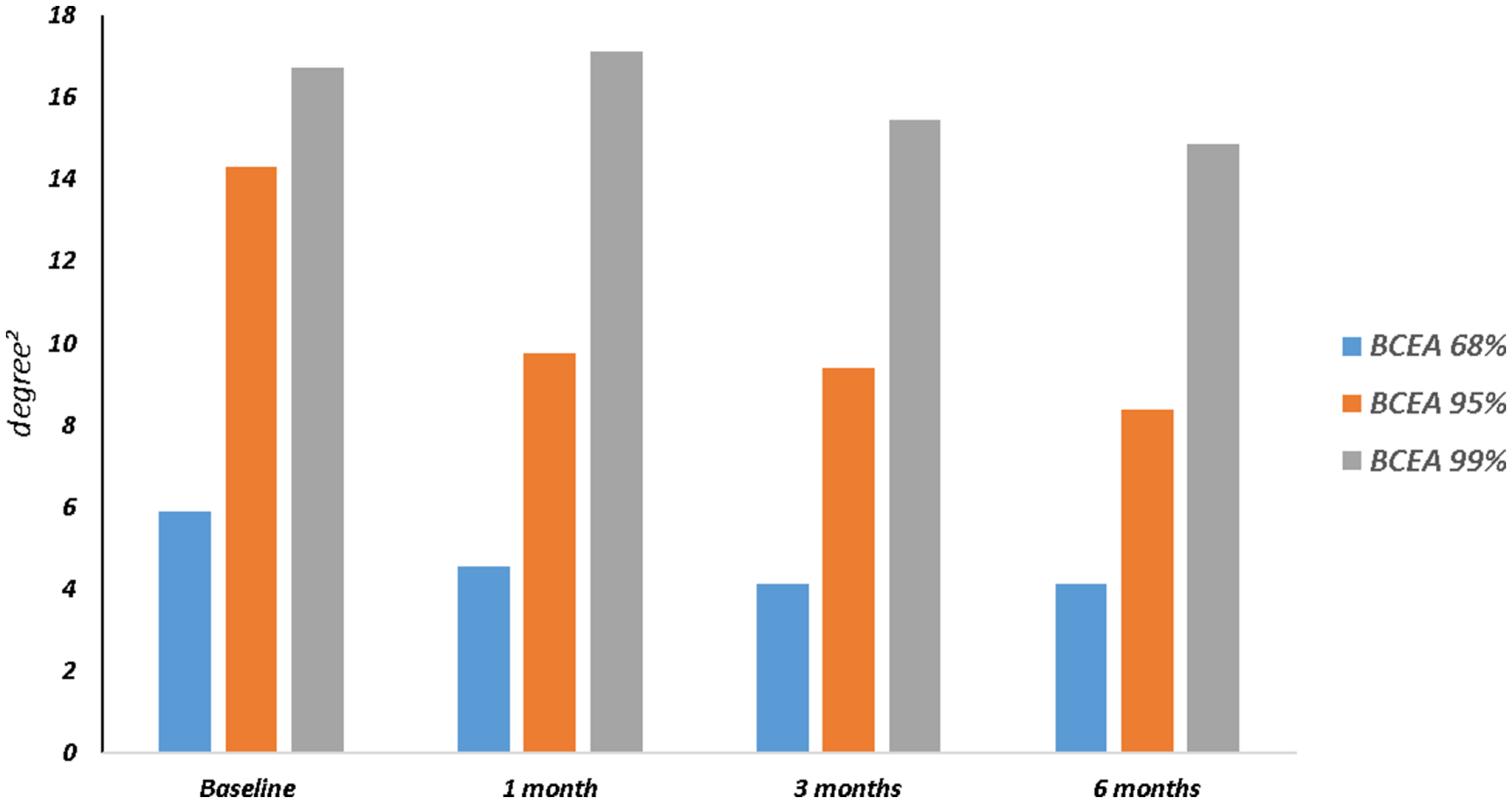
Bivariate contour ellipse area (BCEA) 68.2%, BCEA 95.4% and BCEA 99.6%BCEA changes over follow-up.

The mean RS increased from 11.46 ± 4.91 dB at baseline to 13.29 ± 4.40 dB at 6 months (*p* = 0.003). The mean CRS improved from 8.40 ± 4.76 dB at baseline to 11.24 ± 4.89 dB at the last follow-up (*p* = 0.002). MD changed significantly from −7.69 ± 4.64 dB to −5.99 ± 4.28 dB after 6 months from surgery (*p* = 0.03). The mean value of the BCEA at all different ellipses areas decreased at all time points, but not significantly (BCEA 68.2%, *p* = 0.18; 95.4%, *p* = 0.15; 99.6%, *p* = 0.05; Table 2; Figures 2, 3).

At the last follow-up, 8 (34.8%) patients had worse RS, 7 (30.4%) of these had worse MD than baseline, while only 3 (13.04%) of the 8 patients also had worse CRS. Only 1 (4.3%) patient showed a worsening of CRS alone (Supplementary Figure 2).

**Figure 4:**
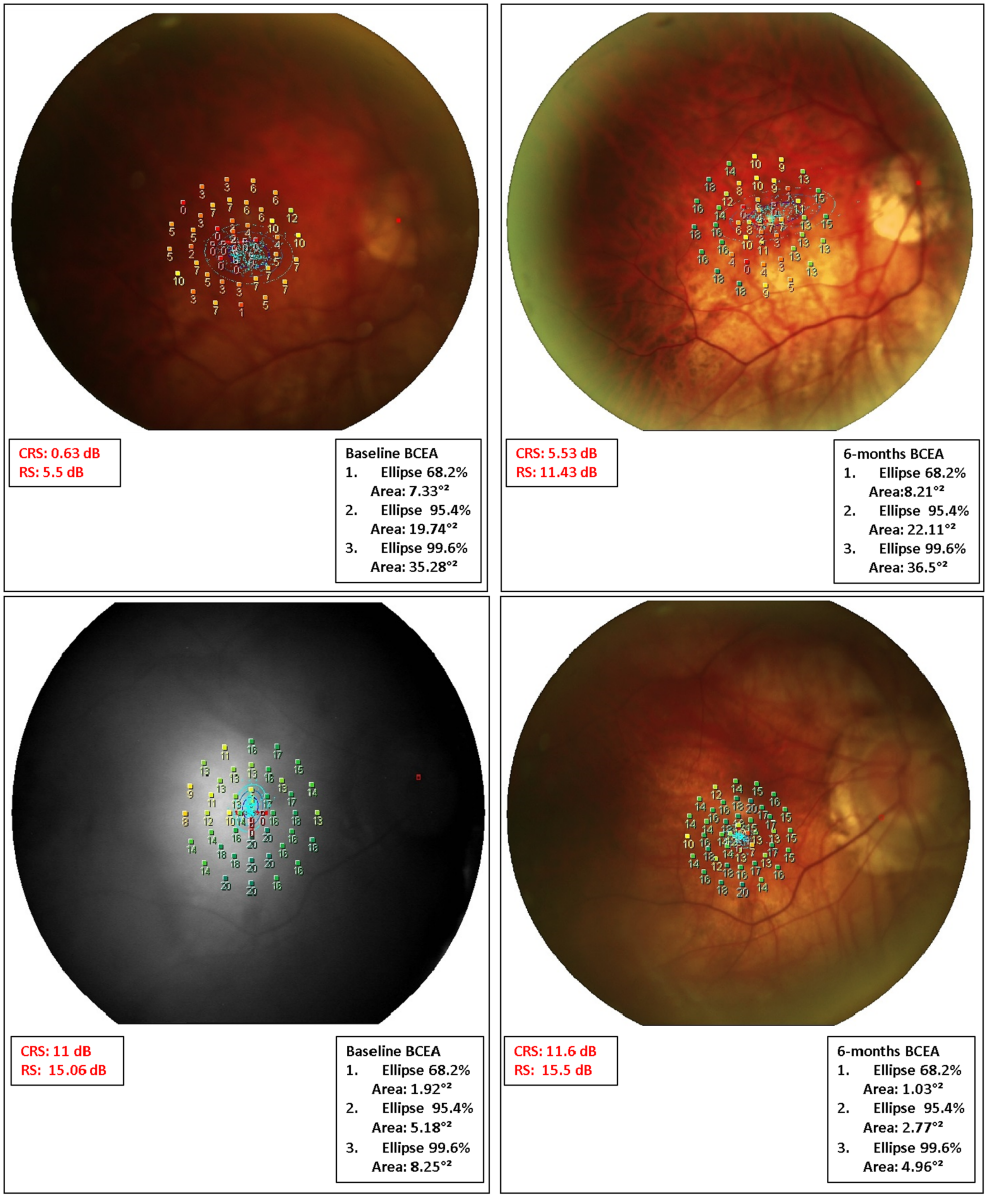
Microperimetric MP1 macular sensitivity interpolate maps at baseline and 6 months after surgery in some representative cases. Case 1. (Top Left) At baseline, microperimetry revealed an absolute scotoma (low values in retinal sensitivity; orange/red) within the central 4 degrees (central 13 points) surrounded by a relative ring scotoma (low-medium values in retinal sensitivity; yellow/orange). (Top Right) After 6 months from surgery, both overall and central sensitivity showed improvement despite low central values. The last BCEA showed a mild enlargement when compared to baseline. However, the majority of the fixation points (blue dots) are still clustered within the central degrees. Case 2. (Bottom Left) Results from the microperimetry test indicate a slight improvement in overall sensitivity from the initial test to the most recent follow-up. Notably, there was a marked improvement in the central scotoma. However, at the paracentral degrees, while some loci showed an increase in sensitivity, many others showed a decrease. The analysis of the BCEA revealed a narrowing of the fixation point cloud, predominantly in the outer ellipses at the last follow-up.

An enlargement of BCEA was reported in a few patients (BCEA 68.2%, 7 patients; BCEA 95.4%, 7 patients; BCEA 99.6%, 7 patients) at the last follow-up compared to baseline (Supplementary Figure 3). Representative cases were reported in Figure 4.

### Phakic vs. pseudophakic eyes

At baseline, pseudophakic eyes had worse functional parameters than phakic ones, but only CRS was significantly worse in pseudophakic patients. The average value of each parameter for each group showed improvement from the baseline to the final follow-up. Only BCVA significantly improved in both groups at all time points. BCEA 95.4% had a significant improvement at all visits in phakic group but only at 6 months in psuedophakic eyes. For the other outcomes, including BCEA 68.2%, RS, MD, and CRS, only in the phakic group a significant improvement was reported over follow-up.

The pseudophakic eyes had a worse mean value of all parameters than phakic ones at all follow-up visits. The mixed model revealed that the combined surgical approach in phakic eyes, involving cataract surgery and inverted ILM-flap technique, was significantly different from the only inverted ILM-flap approach in pseudophakic eyes for several outcomes. The effect of the treatment and time on BCVA and CRS was significantly different between the groups. Only the effect of the interaction between treatment and time on BCVA was significantly different (Table 3).

**Table 3:** Linear mixed model analysis to examine the effect of phakia or pseudophakia on BCVA, BCEA 68.2%, BCEA 95.4%, BCEA 99.6%, RS, MD, and CRS parameters in different times (*n = 23*).

Parameters^*^	Time				*p ^¥^*					
	Pre *(a)*	1 m *(b)*	3 m *(c)*	6 m *(d)*	*b vs (a)*	*c vs (a)*	*d vs (a)*	*c vs (b)*	*d vs (b)*	*d vs (c)*
BCVA										
*Phakic*	0.96 ± 0.20	0.57 ± 0.29	0.41 ± 0.24	0.38 ± 0.26	**<0.001**	**<0.001**	**<0.001**	**0.003**	**0.001**	0.60
*Pseudophakic*	1.03 ± 0.24	0.84 ± 0.28	0.74 ± 0.27	0.68 ± 0.34	**0.02**	**0.001**	**<0.001**	0.22	0.06	0.49
*p ^^^*	0.54	**0.02**	**0.005**	**0.01**						
*Mixed ^§^*	*Treatment* **0.01**	*Time* **<0.0001**	*Interaction* **0.03**							
BCEA 68.2%										
*Phakic*	4.40 ± 5.81	2.85 ± 2.02	2.42 ± 2.20	2.50 ± 2.07	0.08	**0.03**	**0.03**	0.63	0.70	0.93
*Pseudophakic*	9.28 ± 8.49	8.45 ± 8.04	7.99 ± 8.96	7.84 ± 9.41	0.54	0.34	0.29	0.73	0.65	0.91
*p ^^^*	0.05	**0.02**	**0.02**	**0.03**						
*Mixed ^§^*	*Treatment* **0.02**	*Time* 0.13	*Interaction* 0.97							
BCEA 95.4%										
*Phakic*	12.04 ± 15.59	7.14 ± 5.40	6.54 ± 5.97	7.20 ± 5.82	**0.04**	**0.02**	**0.04**	0.80	0.98	0.78
*Pseudophakic*	19.45 ± 19.65	15.72 ± 11.53	15.90 ± 12.60	11.11 ± 12.14	0.30	0.33	**0.02**	0.96	0.21	0.19
*p ^^^*	0.13	0.08	0.06	0.43						
*Mixed ^§^*	*Treatment* 0.08	*Time* **0.02**	*Interaction* 0.61							
BCEA 99.6%										
*Phakic*	14.45 ± 9.54	13.91 ± 10.15	12.15 ± 10.26	12.68 ± 9.58	0.73	0.14	0.26	0.26	0.44	0.73
*Pseudophakic*	21.91 ± 15.27	24.39 ± 13.84	22.96 ± 13.58	19.81 ± 13.11	0.30	0.66	0.38	0.55	0.05	0.19
*p ^^^*	0.14	**0.04**	**0.03**	0.16						
*Mixed ^§^*	*Treatment* 0.06	*Time* 0.22	*Interaction* 0.42							
RS										
*Phakic*	12.46 ± 4.14	13.89 ± 3.15	14.41 ± 3.21	15.06 ± 3.10	**0.03**	**0.003**	**<0.001**	0.42	0.07	0.32
*Pseudophakic*	9.20 ± 6.06	8.43 ± 4.43	9.10 ± 4.51	9.26 ± 4.44	0.43	0.92	0.95	0.49	0.40	0.87
*p ^^^*	0.07	**0.002**	**0.003**	**0.001**						
*Mixed ^§^*	*Treatment* **0.002**	*Time* 0.10	*Interaction* 0.12							
MD										
*Phakic*	−6.93 ± 4.00	−5.71 ± 3.25	−5.14 ± 3.20	−4.52 ± 3.23	0.09	**0.01**	**0.001**	0.43	0.10	0.39
*Pseudophakic*	−9.41 ± 5.82	−9.98 ± 4.05	−8.98 ± 4.81	−9.34 ± 4.69	0.60	0.69	0.95	0.35	0.55	0.74
*p ^^^*	0.16	**0.01**	**0.03**	**0.006**						
*Mixed ^§^*	*Treatment* **0.01**	*Time* 0.16	*Interaction* 0.31							
CRS										
*Phakic*	9.81 ± 4.30	11.02 ± 4.60	12.00 ± 3.84	13.11 ± 3.88	0.08	**0.002**	**<0.001**	0.16	**0.003**	0.11
*Pseudophakic*	5.17 ± 4.39	5.61 ± 3.89	5.88 ± 3.65	6.97 ± 4.41	0.67	0.50	0.09	0.80	0.20	0.30
*p ^^^*	**0.01**	**0.004**	**0.001**	**0.001**						
*Mixed ^§^*	*Treatment* **0.001**	*Time* **0.001**	*Interaction* 0.59							

*As mean and Standard Deviation (M ± SD). BCVA, Best Corrected Visual Acuity; BCEA, Bivariate Contour Ellipse Area which contains 68% or 95% or 99% of fixation points; RS, Retinal Sensitivity; MD, Mean Deviation; CRS, Central Retinal Sensitivity. ^Treatment effect for each time; § Mixed-effects; ^¥^Contrasts of marginal linear predictions.

### Regression model

**Figure 5:**
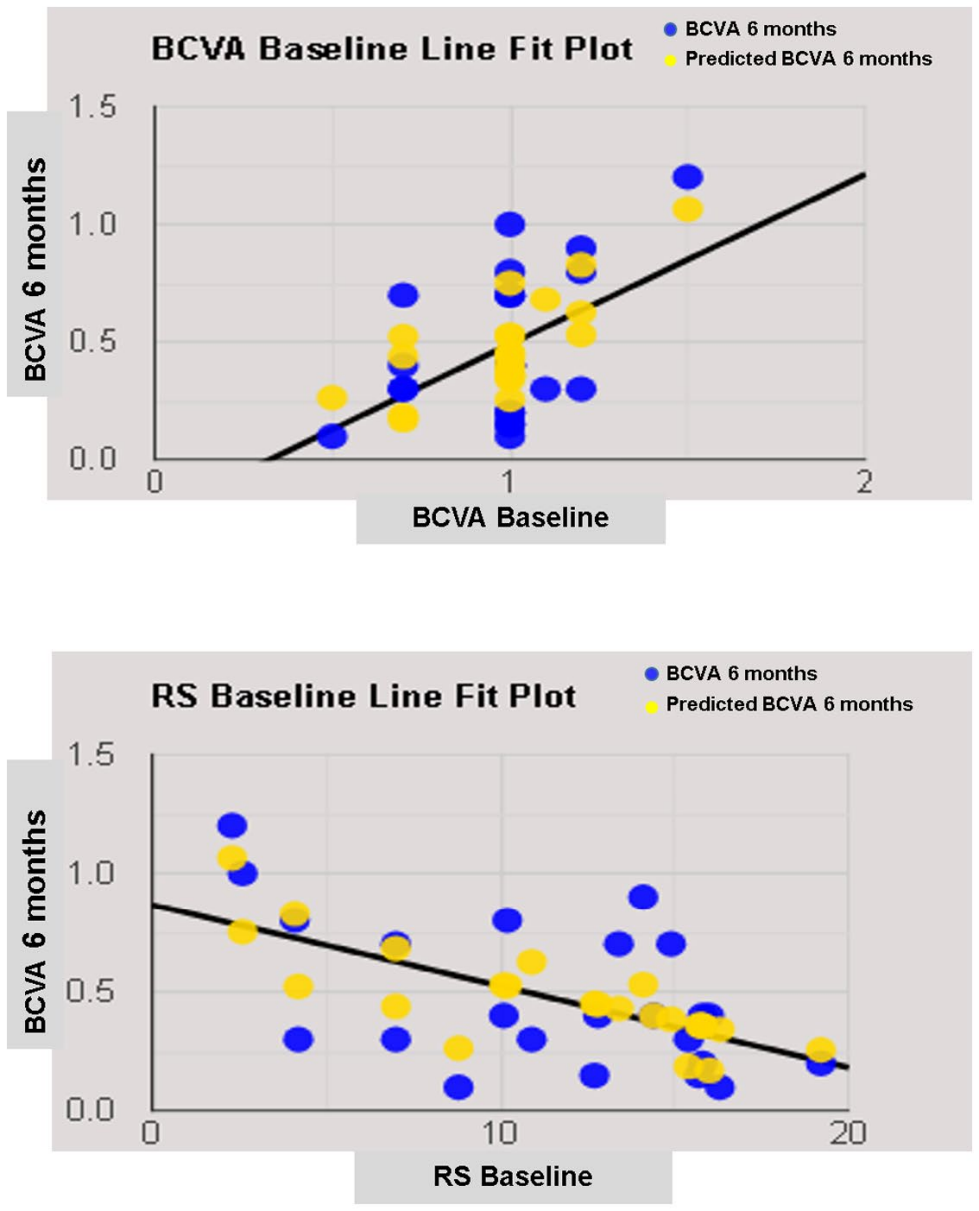
Line fit plot of baseline best corrected visual acuity (BCVA) and retinal sensitivity (RS) as predictive factors of 6-months BCVA.

The simple linear regression model showed that lens status and pre-operative functional parameters, including BCVA, RS, CRS, MD, and BCEA 68.2%, had a significant relationship with 6-month BCVA. Multiple regression models in backward with a stepwise method revealed an independent association of pre-operative BCVA and RS with final BCVA (Table 4; Figure 5).

**Table 4:** Linear regression model of best corrected visual acuity (BCVA) at 6 months on single variables **(A)**. Multiple linear regression model in backward with stepwise method **(B)**.

Parameters	β	se (β)	*p*	C.I. (95%)
(A) BCVA at 6 Months				
Gender	0.13	0.13	0.32	−0.41 to 0.14
Age (yrs)	0.01	0.01	0.31	−0.01 to 0.02
Lens Status	0.30	0.13	0.03	0.03 to 0.57
AL (mm)	0.01	0.03	0.76	−0.06 to 0.08
MH (μm)	0.001	0.0004	0.08	−0.0001 to 0.002
BCVA (Pre)	0.72	0.28	0.02	0.14 to 1.31
RS (Pre)	−0.03	0.01	0.008	−0.06 to −0.01
CRS (Pre)	−0.03	0.01	0.009	−0.06 to −0.01
MD (Pre)	−0.03	0.01	0.01	−0.06 to −0.01
BCEA 68.2% (Pre)	0.019	0.01	0.04	0.0005 to 0.04
BCEA 95.4% (Pre)	0.004	0.004	0.26	−0.004 to 0.01
BCEA 99.6% (Pre)	0.001	0.003	0.65	−0.004 to 0.01
(B) BCVA at 6 Months				
BCVA (Pre)	0.60	0.25	0.02	1.12 to 0.09
RS (Pre)	−0.03	0.01	0.01	−0.007 to −0.05

β, Coefficient; se (β), Standard Error of β; C.I. (95%), Confidential Interval at 95%. AL, axial length; MH, Macular Hole; RS, Retinal Sensitivity; CRS, Central Retinal Sensitivity; MD, Mean Deviation; BCEA, Bivariate Contour Ellipse Area.

## Discussion

In this study, we recorded functional outcomes, including visual acuity, retinal sensitivity, and fixation behavior before and after closure of hMMH over a six-month follow-up. In line with the previous studies (6, 21–23), we found that visual acuity and retinal sensitivity significantly improve after ILM-flap inversion. Good functional results confirmed our choice to use an inverted ILM-flap technique to cover the hole rather than fill the hole, as reported when these two surgical approaches were compared regarding recovery of visual acuity and retinal sensitivity (24). The inverted ILM-flap technique is a promising approach to repair hMMH with a high closure rate (6, 25). The flap acts as a scaffold for the activated Müller cells, promoting the hole-healing process at the macular site (26). Furthermore, the gas tamponade also provides the scaffold or creates a barrier between the retinal pigment epithelium and the fluid while enforcing further stabilization in the inverted flap (27).

After only a month following the surgery, there was an improvement in BCVA for all eyes, with an average gain of 0.32 logMAR. It is worth noting that a significant repair of the outer retinal layers and related visual gain can only be seen by the third month post-surgery (23). So, the removal of cataracts in 69.5% of the eyes alongside vitrectomy may have contributed to the early visual recovery observed. Based on the mixed model analysis, the inverted ILM-flap with cataract surgery had a significantly better effect on BCVA and CRS in phakic eyes compared to the inverted ILM-flap alone in pseudophakic ones. However, the lower pre- and post-operative retinal function observed in pseudophakic eyes can be also related to their larger MH size, which was just associated with a worse visual function (16, 28).

After 6 months, 91.3% of the 23 patients showed an improvement in their visual acuity. It is possible that the selection criteria used in the study, which included the absence of retinal detachment and schisis with hMMH and an axial length over 30 mm in only 3 cases, played a role in the higher rate of visual recovery. This has been reported previously (29–31).

The ILM-flap inversion should allow photoreceptors to assume the correct position during the hole healing and improve visual acuity (22). However, the gliosis process, promoted by the inverted flap (32), could limit or delay the restoration of outer retinal layers (30, 33) and related visual recovery (30, 31). So, the analysis of functional changes after the closure of MH could be underestimated by BCVA, and microperimetry might better analyze those changes (23, 34). Previous studies suggested the necessity of analyzing sensitivity at the parafoveal and foveal site, considering the difference in light sensitivity between different retinal sites, in part due to the “masking effect” of the fixation target during microperimetric test (35, 36) and the different age-related decline in sensitivity between different retinal sites (37). Furthermore, CRS may be more valuable than RS in providing topographic information about retinal sensitivity defects (38). At baseline, microperimetry recorded a lower CRS than RS, revealing a deep central scotoma, which corresponds to the neurosensory defect of the macular hole, surrounded by a relative scotoma around the hole (24, 39). After surgery, the mean value of all parameters of retinal sensitivity (RS, CRS, and MD) improved over 6 months. However, we observed a drop in the last CRS and RS in some patients (9/23; 39%) regardless of their visual acuity improvement. If the sensitivity improvement could be partially related to cataract extraction, as previously observed (40), the ILM peeling before flap reversal could cause paracentral scotomata leading to a reduced sensitivity at central 12° (41) due to temporary swelling of the arcuate nerve fiber layer (20, 21), and the gliosis process, promoted by the inverted ILM-flap, could negatively influence the recovery of retinal sensitivity at central 4 (16, 24, 39). Nevertheless, these surgical effects seem to regress over an extended follow-up (16, 39, 41).

Fixation stability is another functional parameter to be considered, probably more than the fixation location because the fixation site could already be naturally relocated out of the fovea (42). BCEA, as a quantitative parameter of fixation behavior, improved with a reduction in the dimension of the cloud of the fixation points at all follow-ups but failed to reach statistical significance, probably due to the small sample size. Tarita-Nistor et al. observed that patients with MH, whose fixation stability improved the most after ILM peeling, showed the best final visual acuity. In contrast, patients with poorer acuity had the slightest improvement in fixation stability (42). The abnormalities of intraretinal architectural morphology due to the macular hole by itself and its closure by ILM-flap inversion could lead to a new fixation behavior that is not always related to visual acuity, as previously suggested (42).

In linear regression analysis, anatomical parameters such as lens status and pre-operative functional parameters such as BCVA, RS, CRS, MD, and BCEA 68.2% were individually correlated with the last visual acuity. Previous papers on idiopathic MH confirmed the relationship between pre-operative lens status, visual acuity, sensitivity, and fixation stability with final visual acuity (16, 22, 28, 43–45). Unlike previous reports (43, 46), the size of the macular hole did not correlated well with postoperative visual acuity, although the small number of patients analyzed may explain this discrepancy, as previously suggested (47).

A recent paper on hMMH treated with ILM peeling confirmed a relationship between pre- and postoperative visual acuity (48). However, a regression analysis revealing functional predictive factors on visual acuity in hMMH treated with an inverted ILM-flap was not previously performed. We observed a predictive role of pre-operative visual acuity and retinal sensitivity on final visual acuity. Pre-operative visual acuity as a prognostic factor of visual recovery was well known and related to the recovery of the outer retinal layers in idiopathic MH treated with different techniques (22, 28, 45). Also, in high myopic foveoschisis with or without MH, better pre-operative BCVA is a predictor of better visual prognosis (21, 49, 50). Better pre-operative BCVA indicates more remarkable preservation of retinal neuronal function; hence, achieving better visual recovery is more likely after surgery.

On the other hand, the predictive role of retinal sensitivity at 12° confirmed previous results on idiopathic MH (16, 51). We suggested that the inverted ILM-flap not always leads to photoreceptor reconstitution at the foveal site, and retinal sensitivity at 12° is less influenced by foveal microstructure recovery after macular hole closure than sensitivity at central 4 (16).

Although the visual acuity (52), retinal sensitivity, and fixation behavior (53) were linked to the status of the retinal layers in the macular hole condition, the occurrence of functional changes with limited or unremarkable anatomical findings on structural OCT revealed that functional tests are required to solve the shortage of morphological ones. If the analysis of neuroretinal structural parameters, including the diameter of the ellipsoid zone and external limiting membrane defect, their thickness and reflectivity before and after surgery, must be standardized, especially when using image analysis software outside of the OCT device (52), also the microperimetry pays a suboptimal level of accuracy. Firstly, the microperimetric test is influenced by the patient’s clinical condition and their individual “learning effect.” Secondly, the properties of the microperimeter used, including the eye-tracker system and the “Follow-up” program, may not allow for the same level of accuracy when analyzing the fovea versus the perifovea, and may result in suboptimal overlap of each tested point between visits, respectively. Additionally, the test duration, the “ceiling effect,” and the size of the light stimulus can all affect the accuracy of the test. Larger stimuli can involve more photoreceptors that converge on a single ganglion cell (16).

It is important to note that this study has some limitations that should be taken into consideration. These include the fact that it was conducted retrospectively and with a relatively small sample size. Additionally, there was no control group included in the study, and the duration of symptoms was not analyzed. The integrity of retinal layers was not assessed, nor was its relationship with visual function. It is important to take into account the aforementioned inherent variability of the microperimetric test as a limitation when conducting studies.

To our knowledge, this is the first microperimetric analysis of functional changes after the closure of the macular hole in high myopic eyes undergoing the inverted ILM-flap technique.

The effectiveness of the inverted ILM flap technique for functional recovery has been confirmed through improved visual acuity and retinal sensitivity, particularly when combined with cataract extraction. The pre-operative visual acuity, and retinal sensitivity detectable by microperimetry, revealed their predictive role on visual acuity after 6 months from successful hMMH closure by inverted ILM-flap.

## Data availability statement

The raw data supporting the conclusions of this article will be made available by the authors, without undue reservation.

## Ethics statement

The studies involving humans were approved by Eye Clinic, Department of Medical Science, Neuroscience and Sense Organs, University of Bari, Bari, Italy. The studies were conducted in accordance with the local legislation and institutional requirements. The participants provided their written informed consent to participate in this study. Written informed consent was obtained from the individual(s) for the publication of any potentially identifiable images or data included in this article.

## Author contributions

AS: Conceptualization, Writing – original draft. GB: Data curation, Formal analysis, Writing – review & editing. AN: Conceptualization, Formal analysis, Writing – original draft. LL: Data curation, Writing – review & editing. VP: Data curation, Writing – review & editing. VA: Data curation, Writing – review & editing. MP: Data curation, Writing – review & editing. RD: Conceptualization, Formal analysis, Validation, Writing – review & editing. SD: Data curation, Writing – review & editing. PV: Data curation, Writing – review & editing. RB: Data curation, Writing – review & editing. CP: Data curation, Writing – review & editing. TM: Data curation, Writing – review & editing. Eye Clinic Research Group: Data curation, Writing – review & editing. MC: Writing – review & editing. RD’O: Writing – review & editing. FB: Writing – review & editing. GA: Writing – review & editing. GS: Conceptualization, Supervision, Writing – original draft, Writing – review & editing.

## Members of the Eye Clinic Research Group

Antonella Guglielmi, Giacomo Scotti, Marida Gaudiomonte, Roberto Semeraro.
