# Supplementary figures and images for: Microperimetric evaluation and predictive factors of visual recovery after successful inverted internal limiting membrane-flap technique for macular hole in high myopic eyes
Source: Front Med (Lausanne). 2023 Nov 23;10:1276502. doi: 10.3389/fmed.2023.1276502 (PMC10702352; doi:10.3389/fmed.2023.1276502)

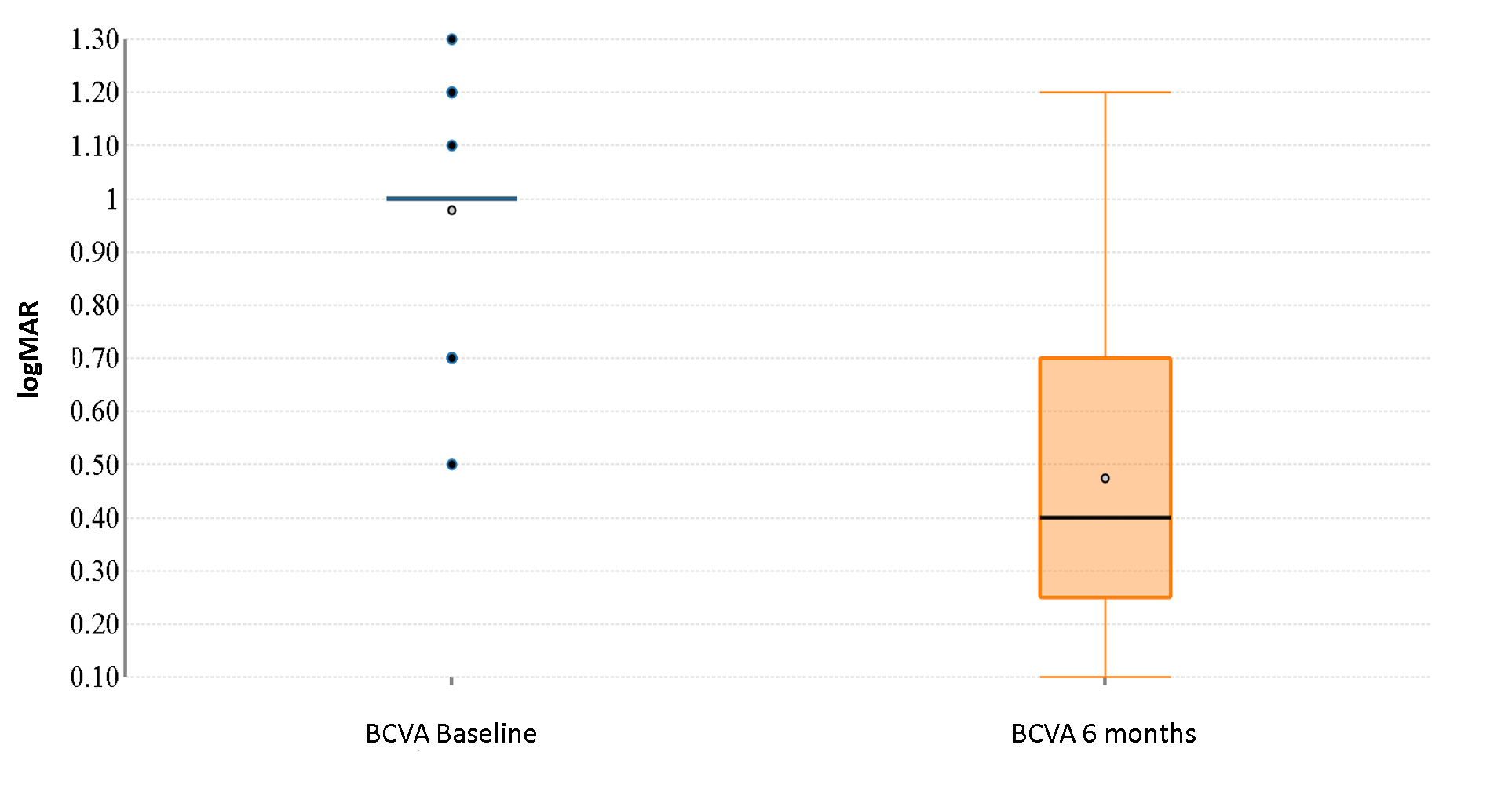

Supplement: Supplementary file 1 [file Image_1.TIFF]

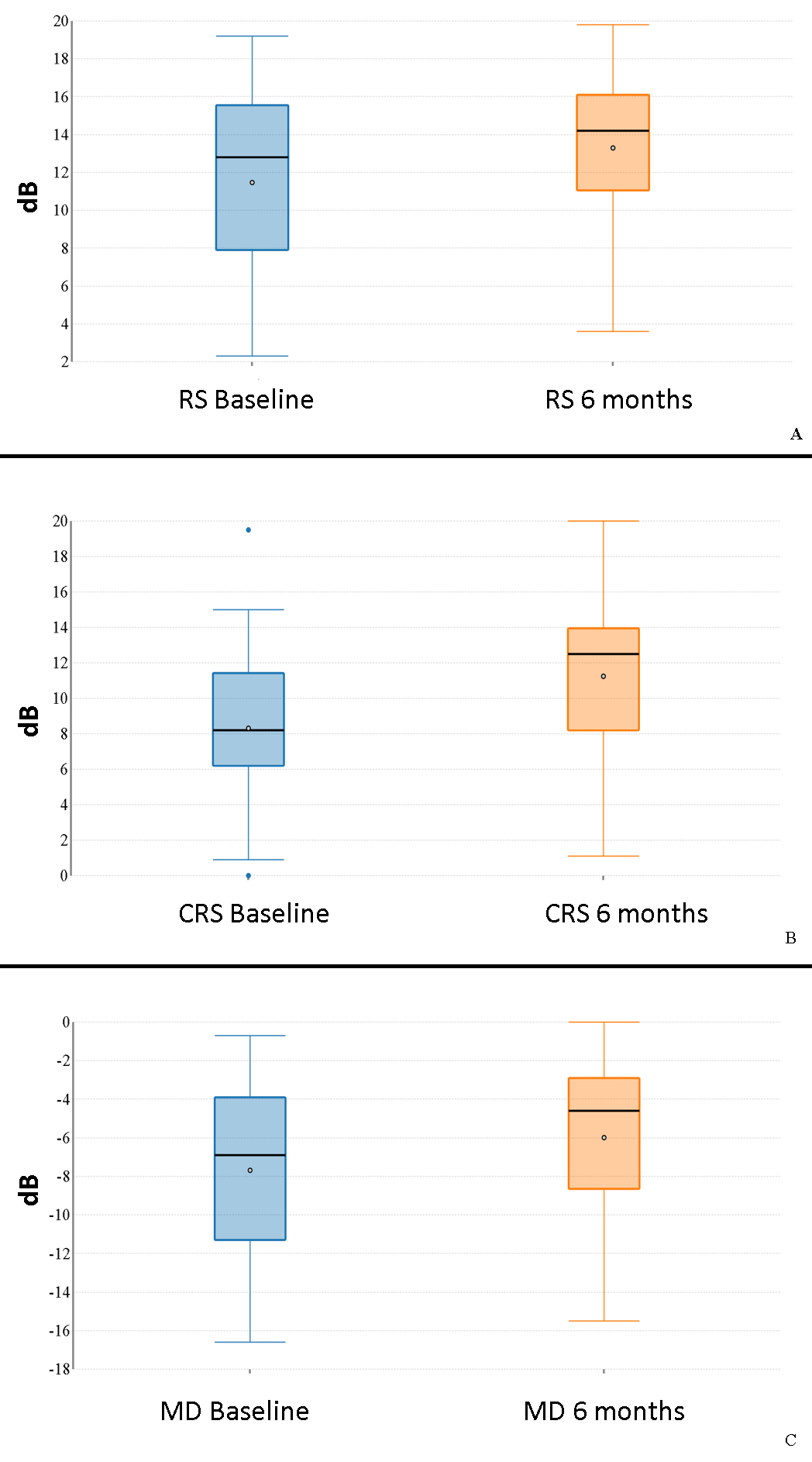

Supplement: Supplementary file 2 [file Image_2.TIFF]

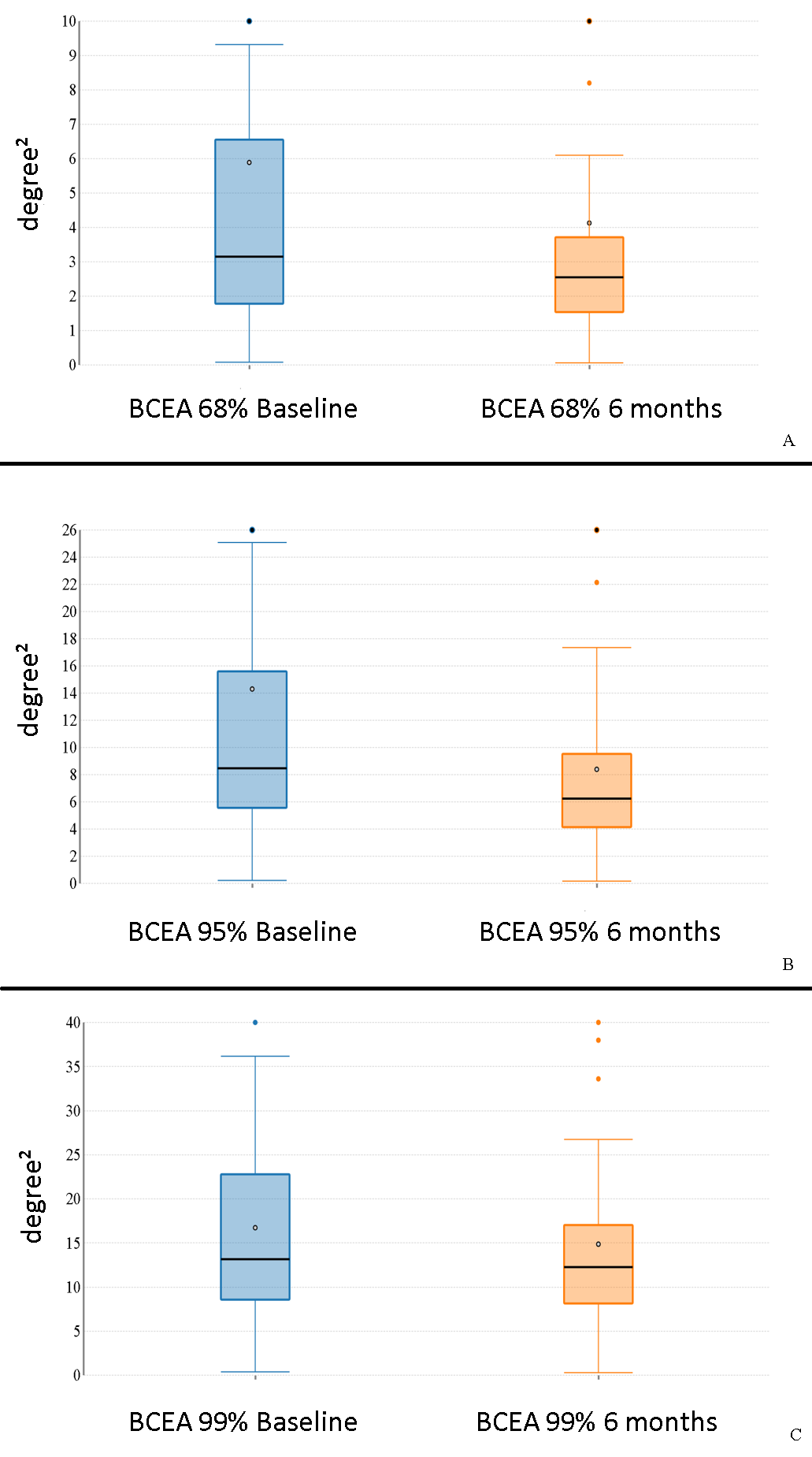

Supplement: Supplementary file 3 [file Image_3.TIFF]
